# Relationship between caregiving burden and alterations in circadian rhythms among spousal caregivers of individuals with cognitive impairment

**DOI:** 10.1186/s12877-025-06316-7

**Published:** 2025-08-23

**Authors:** Shin Young Park, Jung Been Lee, Taek Lee, Ho Yeong Jeong, Sang Yoon Kim, So Yeon Jeon

**Affiliations:** 1 Department of Psychiatry, Chungnam National University Hospital, Daejeon, Republic of Korea; 2 Division of Computer Science and Engineering, College of Engineering, Sunmoon University, Asan, Republic of Korea; 3 Department of Thoracic and Cardiovascular Surgery, Seoul National University Bundang Hospital, Seongnam-si, Gyeonggi-do Republic of Korea; 4 Department of Psychiatry, Seoul Metropolitan Government Seoul National University (SMG-SNU) Boramae Medical Center, Boramaero 5 Gil 20, Seoul, 07061 Republic of Korea

**Keywords:** Caregiving burden, Circadian rhythms of heart rate, Sleep-wake cycle, Spousal caregiver

## Abstract

**Background:**

Caring for individuals with cognitive impairment is demanding and may impact caregiver well-being. This study examined whether caregiving burden is linked to alterations in circadian rhythm of spousal caregivers (SCGs), using both objective and subjective measures.

**Methods:**

A total of 104 SCGs were enrolled, of which 54 wore Fitbit devices to collect objective data on sleep-wake cycles and circadian heart rate rhythm (CHR). Subjective sleep quality was evaluated using the Pittsburgh Sleep Quality Index (PSQI). Multiple regression analyses were conducted to examine the association between caregiving burden, as measured by the Zarit Burden Interview (ZBI), and circadian rhythm variables.

**Results:**

Higher caregiving burden was related to lower goodness of fit (β = − 0.306, t = − 2.144, *p* = 0.037) and greater subjective sleep disturbance (β = 0.203, t = 2.021, *p* = 0.046). Although not statistically significant, a trend toward earlier awakening was observed as the caregiving burden increased, particularly in the SCG of individuals with dementia. No significant associations were found for other variables.

**Conclusions:**

Caregiving burden may negatively influence circadian health in SCGs, especially rhythm regularity. These findings suggest a potential connection between caregiving stress and some aspects of circadian function, emphasizing the need for further research on caregiver burden and circadian function.

**Supplementary Information:**

The online version contains supplementary material available at 10.1186/s12877-025-06316-7.

## Introduction

Caring for individuals with dementia can be highly demanding and have adverse effects on caregivers’ physical or mental well-being [1–4]. Caregiving burden is known to cause health issues in caregivers, including depression [5, 6], cognitive decline [7–10], cardiovascular disease [10, 11], and sleep disturbance [11–14]. These effects can be devastating because the primary caregivers of individuals with dementia are often a spouse caregiver (SCG) [15].

Although most of existing literature has focused on sleep disturbances such as reduced sleep duration or poor sleep quality in caregivers [11–14], there is also interest in alteration in circadian rhythm such as sleep-wake cycle [16] and rest-activity rhythm (RAR) [17, 18], which may provide a more comprehensive understanding of caregivers’ behavioral or physiological functions. Disruption of circadian rhythms was also linked to other health problems. For example, changes in RARs, such as lower amplitude, less robust rhythms, and delayed peak activity times, have been associated with transition to mild cognitive impairment (MCI) or dementia in older adults [19], and delayed phases in RARs have been related to depression in older adults [20]. Additionally, it has been reported that the risk of cardiovascular disease was associated with changes in the sleep-wake cycle [21]. Thus, there is a correlation between negative health outcomes due to caregiving burden and circadian rhythm disruption. Therefore, it is essential to understand the alterations of circadian rhythm that arise from SCGs’ caregiving burden.

Most studies on circadian rhythm changes in SCGs have focused on changes in the sleep-wake cycle and have been limited to subjective assessments using self-report questionnaires, such as the Pittsburgh Sleep Quality Index (PSQI) [22]. These studies’ methods rely on self-reported assessments that are influenced by factors such as emotional state and subjective experience, thereby may limit the ability to determine circadian rhythm changes’ exact pattern [23, 24].

Objective measures of circadian rhythms can use biological markers such as core body temperature [25] or melatonin [26], or behavioral indicators such as sleep-wake cycles and rest-activity rhythms (RARs) using polysomnography or actigraphy. Among these, actigraphy is easier to use than other methods and has been used in many studies on circadian rhythms [27, 28]. For example, Fitbit devices objectively estimate sleep patterns [29, 30], and Heart rate data, including circadian rhythms of heart rate (CHR), can be obtained using photoplethysmography sensors embedded in the device [31, 32]. CHR could complement behavioral indicators of circadian rhythms by providing insight into autonomic nervous systems function [33]. Although different from the traditional RAR, CHR is derived from physiological rhythms and serves as an indirect behavioral indicator of the circadian rhythm system, reflecting physical activity and rest patterns [34, 35]. Despite these advantages, there are limited studies on SCGs using actigraphy such as Fitbit to measure their circadian rhythms.

Based on existing evidence linking caregiver burden with circadian rhythm dysregulation, we investigated whether increased caregiving burden would be associated with alterations in circadian rhythm among SCGs. Specifically, we assessed whether caregiving burden was associated with changes in sleep-wake cycles and CHR. Sleep-wake cycles were measured using both Fitbit (objective) and PSQI (subjective), while CHR was derived solely from Fitbit data. Given the limited prior research using objective tools to examine sleep-wake cycles and CHR in SCGs, we broadly examined multiple circadian rhythm parameters to identify those most sensitive to higher caregiving burden.

Given the limited prior research using objective tools for these circadian rhythms of SCG, rather than focusing on a single marker, we examined multiple circadian rhythm parameters to identify which features may be particularly sensitive to higher caregiving burden. In addition, considering previous studies showing sex-based [1] and care recipient diagnosis [36] differences in caregiving burden, we further assessed whether these variables moderated the observed association. By identifying these unexplored areas, this study aims to deepen our understanding of circadian health in SCGs and inform future interventions to support their well-being.

## Methods

### Study design and participants’ characteristics

This study enrolled the SCGs of individuals who visited the geriatric psychiatry clinic at Chungnam National University Hospital (in South Korea) from May 2020 to August 2023. The inclusion criteria for the study participants were as follows: (1) age between 55 and 90 years; (2) serving as the primary caregiver for the spouse; (3) capable of independent functioning; and (4) no diagnosis of dementia. A total of 104 SCGs were recruited for the study. Of these, 54 caregivers voluntarily agreed to wear a Fitbit device. Participation in Fitbit monitoring for more than two weeks was entirely voluntary. The remaining participants declined to wear Fitbit device due to discomfort with wearing the device, scheduling conflicts, or lack of access to a compatible smartphone for data syncing. Of the 54 SCGs, 30 were caring for spouses diagnosed with dementia, 19 were caring for spouses with MCI, and 5 were caring for spouses with normal cognitive function (CN).

Dementia was diagnosed using the DSM-IV criteria, while MCI met the core clinical criteria recommended by the National Institute on Aging and Alzheimer’s Association guidelines [37]. CN was defined as having a CDR score of 0 and a Mini-Mental State Examination (MMSE) score of 27 or higher [38] and was treated for anxiety disorder or insomnia. The SCGs underwent a comprehensive clinical assessment by experienced neuropsychologists and research nurses. This study did not involve any clinical trials. Clinical trial number: not applicable.

### Circadian rhythm assessment

#### Sleep-wake cycle variable

Participants were instructed to wear the device daily for a minimum of two consecutive weeks. The following objective sleep-wake cycle parameters were obtained from wearable Fitbit: sleep duration, sleep efficiency, and sleep onset and offset times (with onset and offset times expressed in minutes from midnight).

The Korean version of the PSQI [39] was used to determine participants’ self-assessed sleep-wake cycle [22]. In the PSQI, each question is assigned a score ranging from 0 to 3, resulting in a total score between 0 and 21. Participants with scores above 5 were considered to have poor sleep quality. Component scores were derived for subjective sleep quality, sleep latency (i.e., time taken to fall asleep), sleep duration, habitual sleep efficiency (i.e., the ratio of time a person actually sleeps to the total time they spend in bed), sleep disturbances, use of sleep medications, and daytime dysfunction.

#### Circadian rhythms of heart rate parameters

Heart rate data were collected via photoplethysmography sensors embedded in the Fitbit device, summarized every 15 min, and averaged over the next valid day to generate participant-level estimates. If less than 75% of heart rate data was collected during the analysis time, the analysis was excluded. Data were retrieved using a custom Python script via the Fitbit Web API. The utility and feasibility of using Fitbit in clinical research has been previously reported and clinical outcomes have been documented in several studies [29, 31].

To evaluate CHR, we applied cosinor analysis via CosinorPy using the 2-day continuous heart rate data. Cosinor analysis can measure the following key circadian rhythm parameters: amplitude (half the difference between peak and trough of the fitted curve), Midline Estimating Statistic of Rhythm (MESOR; the rhythm-adjusted mean heart rate), acrophase(the timing of the peak in the fitted rhythm, expressed in hours), and goodness of fit (GoF). GoF was calculated as an R-squared value, indicating how well the observed data fit the cosinor model. Values closer to 1 indicate better model fit and stronger circadian rhythmicity.

### Clinical assessments

#### Caregiving burden

Caregiving burden was assessed using the Korean version [40] of 22-item Zarit Caregiver Burden Interview (ZBI). Each item is scored on a five-point Likert scale, ranging from 0 (never) to 4 (nearly always), yielding a total score between 0 and 88 [41].

#### Other clinical variable assessments

Global cognition of each participant was assessed using the Korean version of the MMSE [38]. To evaluate the severity of depressive symptoms, we used the Korean version of the Geriatric Depression Scale [42]. Further, we used the Korean version of the International Physical Activity Questionnaire (IPAQ) [43], which utilizes the Metabolic Equivalent Task (MET) variable to determine physical activity categories. The total minutes spent engaged in physical activity of various intensities over the past seven days were identified, and responses were transformed into MET-minutes per week (MET-min/week) according to the IPAQ scoring protocol [44]. Average MET scores were calculated for each activity type. The following MET values were used: walking = 3.3 MET, moderate-intensity activity = 4.0 MET, vigorous-intensity activity = 8.0 MET. Total physical activity was calculated as the sum of the MET-min/week values derived from walking, moderate-intensity activity, and vigorous-intensity activity.

### Statistical analysis

To investigate whether there were differences in the characteristics of the full sample (*n* = 104) and the Fitbit-wearing subgroup (*n* = 54, of whom 52 also completed the PSQI), Mann–Whitney U tests were used for continuous variables, and chi-square tests were used for categorical variables.

Multiple regression analyses were then conducted to examine the association between caregiving burden, as measured by the ZBI, and circadian rhythm related variables. Analyses were performed sequentially for: (1) Fitbit-derived sleep-wake cycle parameters (sleep duration, sleep efficiency, sleep onset time, and offset time); (2) PSQI scores (total and subdomains); and (3) CHR parameters (amplitude, MESOR, acrophase, and GoF). In all models, SCG’s age and sex, as well as the care recipient’s cognitive status (dementia vs. non-dementia), were included as covariates.

Additional moderation analyses were conducted to examine whether SCG sex or care recipient cognitive status moderated the relationship between caregiving burden and circadian rhythm outcomes. Care recipient cognitive status was categorized as dementia versus non-dementia for these analyses. To limit the number of exploratory tests, these were only performed on sleep or circadian variables that showed a trend-level association (*p* < 0.1) with caregiver ZBI score in the initial regression model. The interaction term ([moderator] × [ZBI]) was used as an independent variable; sex of the SCG, as well as the cognitive status of care recipients, were treated as covariates when appropriate, while circadian rhythm variables were treated as dependent variables.

All analyses were performed using SPSS version 21.0 (SPSS Inc., Chicago, IL). Statistical significance was set at *p* < 0.05.

## Results

### Characteristics of the study population

Table 1 summarizes the characteristics of the full sample (*n* = 104) and the Fitbit subgroup (*n* = 54) who wore the Fitbit.

**Table 1:** Sample characteristics

Variable	Full sample	Fitbit	*p*-value
	*n* = 104	*n* = 54	
SCG factors			
Age, y	72.88 ± 7.07	71.77 ± 6.47	0.254
Sex, F (%)	33.70	33.3	0.968
Education, y	8.93 ± 4.49	9.32 ± 4.34	0.776
Household, n	1.16 ± 0.36	1.08 ± 0.38	0.140
BMI	24.68 ± 3.45	24.44 ± 2.92	0.853
Other disease (%)			
DM	23.10	20.8	0.718
HTN	52.90	41.5	0.160
Stroke	3.80	3.8	0.973
Hyperlipidemia	39.40	49.1	0.269
TIA	-	-	-
Coronary diseases	8.70	5.7	0.494
APOE4 positive	15.40	21.3	0.756
MMSE	24.95 ± 2.91	25.77 ± 2.24	0.172
GDS	12.90 ± 7.07	11.98 ± 6.40	0.273
ZBI	34.99 ± 19.75	33.75 ± 18.13	0.562
IPAQ total	2439.74 ± 3159.75	1807.35 ± 2429.22	0.561
PSQI total	8.25 ± 3.56	7.84 ± 3.42	0.476
Subjective sleep quality	1.21 ± 0.63	1.23 ± 0.47	0.854
Sleep latency	2.52 ± 0.71	2.52 ± 0.61	0.684
Sleep duration	1.10 ± 1.05	0.96 ± 0.97	0.462
Habitual sleep efficiency	1.19 ± 1.30	1.00 ± 1.10	0.492
Sleep disturbance	1.42 ± 0.62	1.29 ± 0.50	0.135
Use of sleep medicine	0.63 ± 0.10	0.52 ± 1.02	0.521
Daytime dysfunction	0.95 ± 1.46	0.59 ± 0.60	0.571
Care-recipient factor			
MMSE	19.46 ± 5.90	20.21 ± 5.35	0.779
CDR	0.82 ± 0.55	0.77 ± 0.44	0.482
NPI	20.98 ± 19.68	15.96 ± 16.64	0.095
Cognitive status of care-recipients (%)			
Dementia	59.6	55.6	0.624
Non-dementia	40.4	44.4	0.624

Values are given as means ± standard deviations

*Abbreviations:* *BMI* Body mass index, *DM* Diabetes Mellitus, *HTN* Hypertension, *TIA* Transient ischemic attack, *APOE4* Apolipoprotein ε4, *MMSE* Mini-Mental State Examination, *GDS* Geriatric Depression Scale, *ZBI* Zarit Burden Interview, *IPAQ* International Physical Activity Questionnaire, *PSQI* Pittsburgh Sleep Quality Index, *CDR* Clinical Dementia Rating, *NPI* Neuropsychiatric Inventory

All SCGs included in the study were living with the care recipient at the time of participation. For the Fitbit group (*n* = 54), participants wore actigraphy for an average of 12.02 days (SD = 4.92). 87% of participants provided at least 7 valid days of data, ensuring adequate data quality for circadian rhythm analysis. No statistically significant differences in demographic characteristics were observed between the two groups. Both groups had total PSQI scores exceeding the clinical cut-off point of 5, with respective scores of 8.25 ± 3.56 and 7.84 ± 3.42. Similarly, although the ZBI is usually considered a continuous scale, the observed total scores in both groups (34.99 ± 19.75 and 33.75 ± 18.13) suggest relatively high levels of caregiver burden compared to previous studies [45].

We also performed a comparative analysis of Fitbit-derived sleep-wake parameters, PSQI, and CHR parameters categorized by care recipients’ cognitive status (dementia vs. non-dementia) within the Fitbit group, but no significant differences were found (Additional file 2).

### Association between caregiving burden and fitbit-derived sleep-wake parameters

Although no statistically significant associations were found between caregiving burden and Fitbit-derived sleep-wake cycle parameters, there was a trend decreasing offset time as caregiving burden increased (β = − 0.254, t = − 1.725, *p* = 0.091) (Table 2).

**Table 2:** Association between caregiving burden and Fitbit-derived sleep-wake parameters among SCGs (*n* = 54)

	ZBI of SCGs			
*Sleep-wake cycle variable*	***β***	***SE***	***t***	***p*** ^***a***^
Sleep duration(min)	−0.193	0.519	−1.307	0.197
Sleep efficiency(%)	0.040	0.000	0.266	0.791
Onset time(min)	−0.107	0.938	−0.708	0.483
Offset time(min)	−0.254	0.778	−1.725	0.091

Covariates of the linear regression model included SCGs’ age and sex, as well as care recipients’ cognitive status

*Abbreviations:* *SCG* Spousal caregiver, *ZBI* Zarit Burden Interview

### Association between caregiving burden and subjective sleep outcomes (PSQI)

To better understand overall trends in caregivers’ subjective sleep outcomes, we examined the relationship between PSQI and caregiving burden using the full sample (*n* = 104). As the caregiving burden increased, sleep disturbance worsened significantly (β = 0.203, t = 2.021, *p* = 0.046), while sleep latency exhibited a trend-level association that did not reach the conventional threshold for statistical significance (β = 0.196, t = 1.953, *p* = 0.054) (Table 3).

**Table 3:** Association between caregiving burden and PSQI of SCGs (*n* = 104)

	ZBI of SCGs			
*PSQI score*	***β***	***SE***	***T***	***p***
PSQI total	0.136	0.018	1.351	0.180
Subjective sleep quality	0.089	0.003	0.875	0.384
Sleep latency	0.196	0.003	1.953	0.054
Sleep duration	−0.057	0.005	−0.569	0.570
Habitual sleep efficiency	0.100	0.007	0.996	0.322
Sleep disturbance	0.203	0.003	2.021	0.046*
Use of sleep medicine	−0.122	0.006	−1.106	0.271
Daytime dysfunction	0.160	0.007	1.598	0.113

Covariates of the linear regression model included age and sex of SCGs, as well as the cognitive status of care-recipients

*Abbreviations:* *SCG* Spousal caregiver, *ZBI* Zarit Burden Interview, *PSQI* Pittsburgh Sleep Quality Index

*p < 0.05

However, no statistically significant associations were found between caregiving burden and PSQI total or subdomain scores among Fitbit wearers (*n* = 54) (Additional File 1).

### Association between caregiving burden and circadian heart rate rhythm (CHR)

**Fig. 1:**
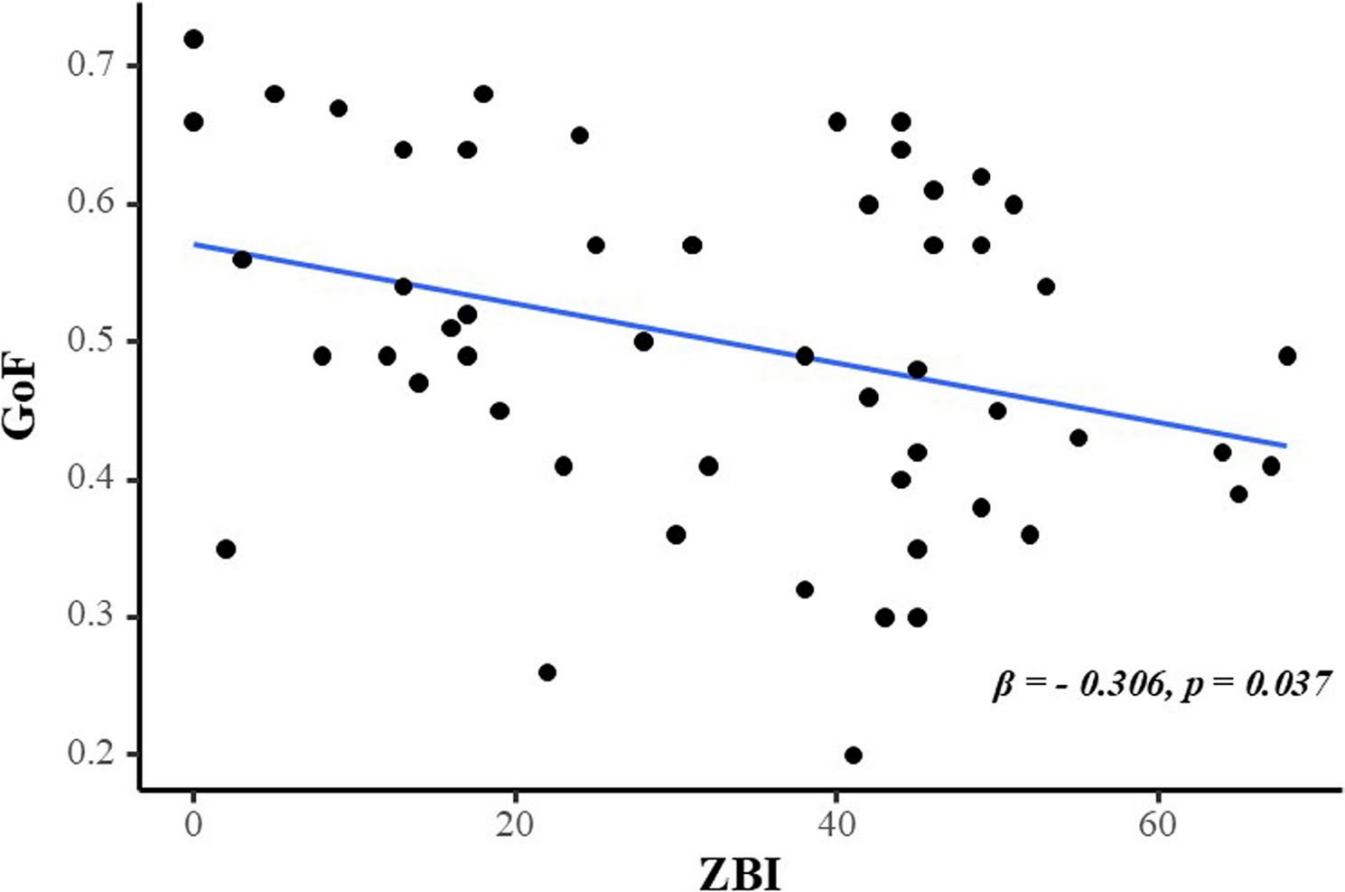
Association between caregiving burden and SCGs’ GoF. Notes: Covariates of the linear regression model include age and sex of SCGs and the cognitive level of care-recipients. Abbreviations: SCG, spousal caregiver; ZBI, Zarit Burden Interview; GoF, goodness of fit

An association was observed between increased caregiving burden and lower GoF values (β = − 0.306, t = − 2.144, *p* = 0.037; Table 4), indicating that caregivers’ CHRs became less regular as burden increased (Fig. 1).

**Table 4:** Association of caregiving burden and CHR parameters (*n* = 54)

	ZBI of SCGs			
CHR parameters	β	SE	t	*p*^a^
Amplitude	−0.180	0.020	−1.255	0.215
MESOR	0.038	0.057	0.265	0.792
GoF	−0.306	0.001	−2.144	0.037*
Acrophase	−0.004	0.011	−0.027	0.978

Covariates of the linear regression model included age and sex of SCGs, as well as the cognitive status of care-recipients

*Abbreviations:* *SCG* Spousal caregiver, *ZBI* Zarit Burden Interview, *CHR* Circadian rhythm of heart rate, *MESOR* Midline Estimating Statistic of Rhythm, *GoF* Goodness of fit

*p < 0.05

Other parameters related to the CHRs did not exhibit a significant association with the ZBI score.

### Moderating effects of SCG sex and care recipient cognitive status on offset time and GoF

**Fig. 2:**
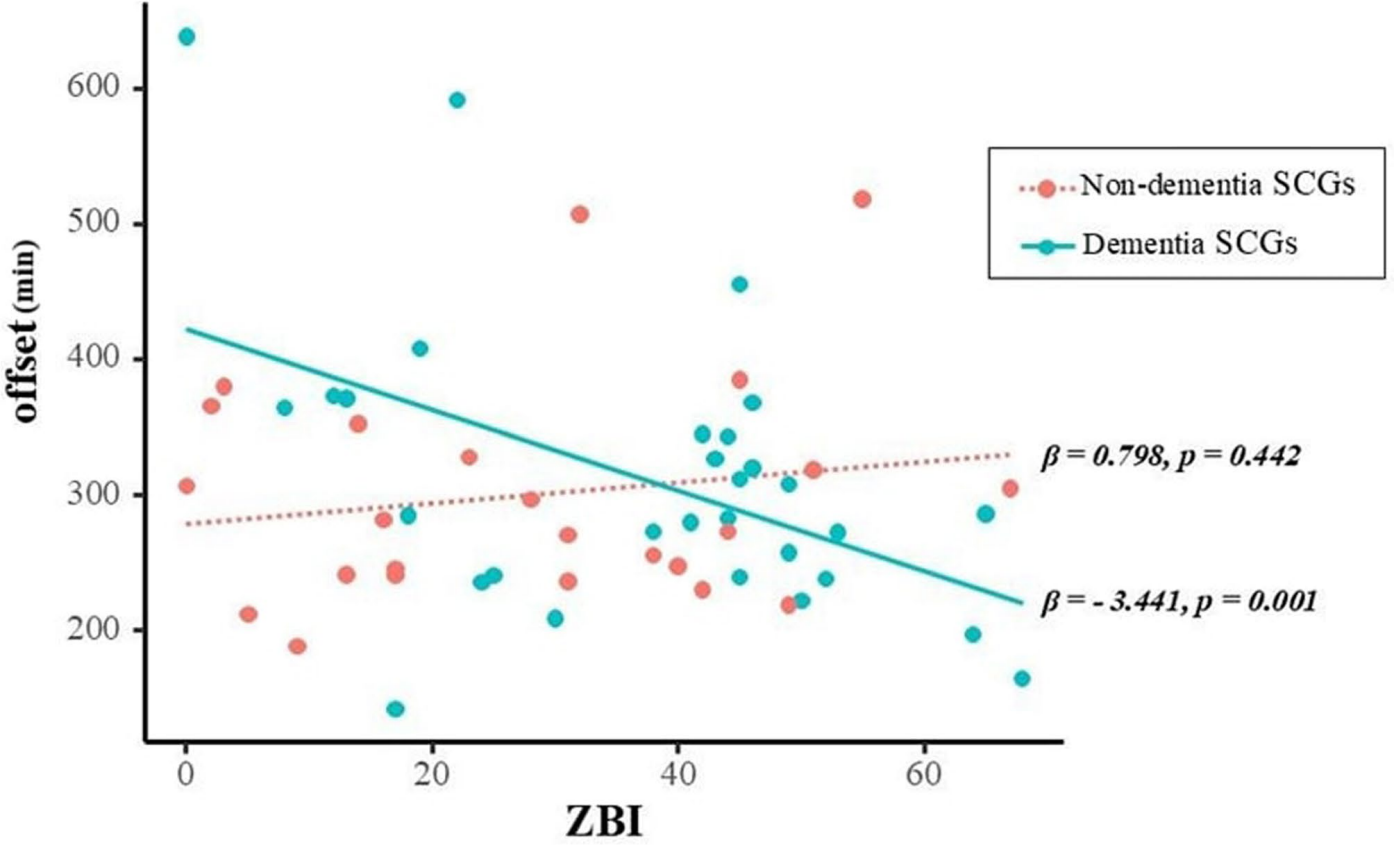
Moderating effect of cognitive level of care recipients and SCGs’ GoF. Notes: Covariates include SCGs’ age and sex. Abbreviations: GoF, goodness of fit; SCG, spousal caregiver; ZBI, Zarit Burden Interview

To further explore potential moderator effects of SCGs’ sex and care recipients’ cognitive status, we conducted additional interaction analyses exclusively for variables with a p-value below 0.1 (off-set time and GoF) in the primary regression models. The care recipients’ cognitive status significantly moderated the effect of caregiving burden on offset time (β = −4.239, t = −2.924, *p* = 0.005) (Table 5). Further subgroup analyses revealed a significant correlation between heightened caregiver burden and earlier awakening among SCGs of individuals with dementia (β[SE] = −3.441[1.020], t = −3.375, *p* = 0.001), whereas no such association was observed among SCGs of individuals without dementia (β[SE] = 0.798[1.030], t = 0.775, *p* = 0.442) (Fig. 2).

**Table 5:** Moderating effect of sex of SCGs and cognitive status of care recipients on the association between caregiving burden versus offset time and GoF of SCGs

Dependent variable	Moderator	Independent variable	B (LLCI-ULCI)	t	*p*
*Offset time*					
	Sex	ZBI	−0.877 (−2.659–0.906)	−0.989	0.328
		Sex	52.499 (−68.888–173.885)	0.870	0.389
		ZBI x Sex	−1.993 (−5.676–1.691)	−1.088	0.282
	Cognitive status	ZBI	0.798 (−1.272–2.868)	0.775	0.442
		Cognitive level	164.708 (55.188–274.227)	3.024	0.004
		ZBI x cognitive level	−4.239 (−7.154–−1.325)	−2.924	0.005*
*GoF*					
	Sex	ZBI	−0.001 (−0.003–0.001)	−1.090	0.281
		Sex	0.136 (−0.010–0.282)	1.874	0.067
		ZBI x Sex	−0.004 (−0.008–0.001)	−1.711	0.094
	Cognitive status	ZBI	−0.002 (−0.004–0.001)	−1.238	0.222
		Cognitive status	0.018 (−0.0128–0.163)	0.247	0.806
		ZBI x cognitive status	−0.001(−0.005–0.003)	−0.362	0.719

Moderate for sex effect adjusted for age of SCGs and cognitive level of care-recipient

Moderate for cognitive level of care recipients adjusted for age and sex of SCGs

*Abbreviations:* *SCG* Spousal caregiver, *ZBI* Zarit Burden Interview, *GoF* Goodness of fit

## Discussion

While prior studies have examined circadian rhythm disruption caregivers, relatively few have explored alterations in circadian rhythm using objective metrics such as sleep-wake cycle and CHR, and even fewer have focused specifically on SCGs. We found that greater caregiving burden was significantly associated with reduced circadian rhythm regularity of heart rate, as reflected by lower GoF in CHR. In addition, caregiving burden was related to greater subjective sleep disturbance and, although not statistically significant, showed a tendency toward earlier awakening, particularly among SCGs of individuals with dementia.

Among the investigated circadian rhythm parameters, GoF was the only indicator significantly associated with caregiving burden. GoF reflects the overall regularity of circadian rhythms, with lower values indicating a more disorganized or unstable rhythm pattern [46]. This finding suggests that caregiving stress may disrupt the general organization of physiological rhythms rather than affecting specific parameters such as acrophase or amplitude. This is consistent with prior RAR-based studies, which reported that caregivers who are unable to leave individuals with dementia alone often experience frequent nighttime awakenings, potentially contributing to rhythm fragmentation [18]. However, unlike those findings—where MESOR was also affected—our results suggest that caregiving burden may primarily influence rhythm regularity. This difference may reflect the nature of CHR, which captures not only behavioral rhythms, but also internal physiological stress responses mediated by autonomic regulation [33–35].

Although offset time was not significantly associated with caregiving burden, a trend toward earlier awakening was observed, particularly among SCGs of individuals with dementia. This may reflect the tendency of these caregivers to wake up early and struggle to return to sleep due to care recipients’ nocturnal behaviors [47]. This multifactorial interaction may help explain why the trend toward earlier awakening was more pronounced in this subgroup. However, prior studies suggest that such disruptions are not solely attributable to the care recipient but also caregivers’ factors [12, 47–49]. In our study, SCGs reported poorer subjective sleep quality despite sufficient sleep duration, as indicated by higher PSQI global and subdomain scores (excluding sleep duration) compared to cognitively healthy older adults in Korea [50], as well as a positive association between caregiving burden and subjective sleep disturbance. This finding contrasts with that of Ryuno et al. [14], who reported a significant association between caregiving burden and reduced sleep duration. One possible explanation for this discrepancy is the use of different devices: the ActiGraph GT9X used in Ryuno’s study is known to underestimate sleep duration, whereas Fitbit has shown greater concordance with PSG in detecting sleep parameters [51]. Additionally, the caregivers in our study were older (mean age: 70.2 vs. 66.9 years) and had higher PSQI scores (8.25 ± 3.56 vs. 5.2 ± 3.6), suggesting greater baseline sleep disturbance that may have masked any association between caregiving burden and sleep duration. Although neither result reached statistical significance, Fitbit data showed a trend toward earlier awakening, and PSQI responses indicated longer sleep latency with increasing caregiving burden. These discrepancies may reflect differences in assessment tools, or the varied ways in which caregiving stress manifests in individual sleep patterns. For instance, caregivers often manage dual responsibilities (e.g., medication schedules, hospital visits), which may delay sleep onset [12], while nighttime vigilance or anticipatory anxiety may lead to earlier awakening [47].

Taken together, these findings imply that increased caregiving burden may lead to changes in the sleep-wake cycle—not necessarily through reduced sleep duration, but via shifts in sleep timing that may be either advanced or delayed, depending on individual adaptation. More critically, it is not the specific timing (e.g., waking early or sleeping late) that may matter most, but rather the disruption in regularity. Additionally, such disruptions may persist even after care recipients’ nocturnal behaviors improve, implying that alterations in caregivers’ circadian regulation may become chronic [47]. Therefore, when assessing SCGs’ sleep problems, it is crucial to consider other aspects of sleep quality beyond sleep duration. Specifically, given our results, examining the robustness of circadian rhythms in caregivers with a high caregiving burden may be particularly beneficial.

Alterations in circadian rhythms have been associated with depression [18] and cognitive decline in caregivers [19]. Disruptions to the circadian system can impact cardiovascular health independently of behavioral and environmental cycles [11, 21]. These disruptions are important because they can cause serious practical problems for caregivers, such as caregivers failing to recognize new symptoms, forgetting to administer medication, being unable to multitask, and struggling to make rational decisions [12]. Studies have shown that circadian rhythm alterations in caregivers can be improved with appropriate sleep interventions, thus highlighting the importance of early intervention [12, 14].

## Strengths and limitations

This study has several strengths, including its use of objective measurements to examine circadian rhythms and its specific focus on SCGs. While CHR is distinct from traditional RAR, it provides unique physiological insights into circadian regulation by primarily reflecting autonomic nervous system activity, thereby complementing behavioral indicators [33–35]. Moreover, we assessed multiple circadian rhythm variables, including those derived from both sleep-wake cycles and CHR. Although not all variables showed statistically significant associations, the consistent directionality and conceptual coherence of the findings support the relevance of the selected domains to caregiving burden.

Despite these strengths, several limitations should be acknowledged. Although vascular risk factors were assessed and statistically controlled for, we did not collect detailed information on medications that may affect heart rate, such as beta-blockers, which remains a clear limitation. Furthermore, the absence of heart rate variability (HRV) analysis is notable. The Fitbit device used in this study does not provide access to raw inter-beat interval data through its API, offering only summary-level HRV metrics during sleep periods [52]. This restricted our ability to examine autonomic nervous system activity in more depth. Future studies employing devices capable of high-resolution inter-beat interval recording could allow for a more comprehensive assessment of caregivers’ autonomic and circadian profiles. Additionally, we did not account for non-circadian factors that may influence circadian rhythms, such as voluntary physical activity or lifestyle habits. The relatively small sample size and the inclusion of SCGs of individuals who were CN may also limit the generalizability of the findings. For the interaction analysis, these participants were grouped with non-dementia care recipients group. But the subgroup of SCGs of individuals with CN was particularly small (*n* = 5). While this grouping supported statistical feasibility, comparisons between SCGs of individuals with dementia and without dementia should be interpreted with caution. Lastly, the study may be subject to selection bias, as participants were recruited from a single geriatric psychiatric clinic. This may limit the applicability of the findings to other settings or more diverse caregiving populations.

## Conclusion

Among the circadian rhythm parameters examined, only GoF—a marker of rhythm regularity—was significantly associated with caregiving burden. This suggests that caregiving stress may contribute to disruptions in the stability of physiological rhythms. Higher caregiving burden was also associated with greater subjective sleep disturbance. While offset time showed a non-significant trend, especially among SCGs of individuals with dementia, no other circadian parameters demonstrated notable associations. These findings underscore the potential value of GoF as an objective indicator of circadian rhythm disruption in caregivers. Further research is needed to validate these findings in larger samples and to explore interventions aimed at maintaining circadian health in this population.

## Supplementary Information

Additional file 1. Regression of the caregiving burden versus PSQI subdomain of SCGs (*n*=54). This table shows the results of a regression analysis between caregiving burden (measured by the Zarit Burden Interview) and various subdomains of sleep quality (assessed by the Pittsburgh Sleep Quality Index) in spousal caregivers.
Additional file 2. Characteristics of Fitbit-derived sleep and circadian rhythm heart rate parameters by care recipient cognitive status (dementia vs. non-dementia). This table shows the results of comparative analysis of sleep and circadian rhythm parameters categorized by care recipients’ cognitive status (dementia vs. non-dementia caregivers) within the Fitbit group (*n*=54).

## Data Availability

The data generated and analyzed during the current study are not publicly available due to privacy restrictions but are available on reasonable request from the corresponding author.

## Abbreviations

CHR: Circadian rhythms of heart rate
CN: Cognitively normal
GoF: Goodness of fit
HRV: Heart rate variability
IPAQ: International physical activity questionnaire
MCI: Mild cognitive impairment
MET: Metabolic equivalent of task
MMSE: Mini-mental state examination
MESOR: Midline estimating statistic of rhythm
PSQI: Pittsburgh sleep quality index
SCG: Spousal caregiver
ZBI: Zarit caregiver burden interview

## References

[1] Vitaliano PP, Zhang J, Scanlan JM. Is caregiving hazardous to one’s physical health?? A meta-analysis. Psychol Bull. 2003;129:946–72. 10.1037/0033-2909.129.6.946. 14599289 10.1037/0033-2909.129.6.946

[2] Sabatini S, et al. Health conditions in spousal caregivers of people with dementia and their relationships with stress, caregiving experiences, and social networks: longitudinal findings from the IDEAL programme. BMC Geriatr. 2024;24(1): 171. 10.1186/s12877-024-04707-w. 38373905 10.1186/s12877-024-04707-w PMC10875834

[3] Brodaty H, Donkin M. Family caregivers of people with dementia. Dialogues Clin Neurosci. 2009;11(2):217–28. 10.31887/DCNS.2009.11.2/hbrodaty. 19585957 10.31887/DCNS.2009.11.2/hbrodaty PMC3181916

[4] Koca E, Taşkapilioğlu Ö, Bakar M. Caregiver burden in different stages of Alzheimer’s disease. Noro Psikiyatri Arsivi. 2017;54(1):82–6. 10.5152/npa.2017.11304. 28566965 10.5152/npa.2017.11304 PMC5439478

[5] Beeson RA. Loneliness and depression in spousal caregivers of those with Alzheimer’s disease versus non-caregiving spouses. Arch Psychiatr Nurs. 2003;17(3):135–43. 10.1016/s0883-9417(03)00057-8. 12840806 10.1016/s0883-9417(03)00057-8

[6] Cuijpers P. Depressive disorders in caregivers of dementia patients: a systematic review. Aging Ment Health. 2005;9(4):325–30. 10.1080/13607860500090078. 16019288 10.1080/13607860500090078

[7] von Känel R, et al. Increased Framingham coronary heart disease risk score in dementia caregivers relative to non-caregiving controls. Gerontology. 2008;54(3):131–7. 10.1159/000113649. 18204247 10.1159/000113649

[8] Richardson TJ, Lee SJ, Berg-Weger M, Grossberg GT. Caregiver health: health of caregivers of Alzheimer’s and other dementia patients. Curr Psychiatry Rep. 2013;15(7): 367. 10.1007/s11920-013-0367-2. 23712718 10.1007/s11920-013-0367-2

[9] Vitaliano PP, Murphy M, Young HM, Echeverria D, Borson S. Does caring for a spouse with dementia promote cognitive decline?? A hypothesis and proposed mechanisms. J Am Geriatr Soc. 2011;59(5):900–8. 10.1111/j.1532-5415.2011.03368.x. 21568959 10.1111/j.1532-5415.2011.03368.x

[10] Xu XY, Kwan RYC, Leung AYM. Factors associated with the risk of cardiovascular disease in family caregivers of people with dementia: a systematic review. J Int Med Res. 2020;48(1): 300060519845472. 10.1177/0300060519845472. 31115265 10.1177/0300060519845472 PMC7140198

[11] Bos T. Sleep and cardiovascular disease in Alzheimer’s caregivers: An examination of cross-sectional and longitudinal associations and potential treatment response, UC San Diego, 2021. Available: https://escholarship.org/uc/item/3xs8q0hm. Accessed 22 Mar 2024.

[12] Gao C, Chapagain NY, Scullin MK. Sleep duration and sleep quality in caregivers of patients with dementia: a systematic review and meta-analysis. JAMA Netw Open. 2019;2(8):e199891. 10.1001/jamanetworkopen.2019.9891. 31441938 10.1001/jamanetworkopen.2019.9891 PMC6714015

[13] Leggett AN, Morley M, Smagula SF. “‘It’s Been a Hard Day’s Night’: sleep problems in caregivers for older adults,.” Curr Sleep Med Rep. 2020;6(1):1–10. 10.1007/s40675-020-00164-0. 34079689 10.1007/s40675-020-00164-0 PMC8168632

[14] Ryuno H, et al. Association between sleep, care burden, and related factors among family caregivers at home. Psychogeriatrics. 2020;20(4):385–90. 10.1111/psyg.12513. 31975544 10.1111/psyg.12513 PMC7496993

[15] Hu B, Shin P, Han E, Rhee Y. Projecting Informal Care Demand among Older Koreans between 2020 and 2067. Int J Environ Res Public Health. 2022;19(11):6391. 10.3390/ijerph19116391. 35681979 10.3390/ijerph19116391 PMC9180884

[16] Dhruva A, et al. Sleep-wake circadian activity rhythms and fatigue in family caregivers of oncology patients. Cancer Nurs. 2012;35(1):70–81. 10.1097/NCC.0b013e3182194a25. 21760489 10.1097/NCC.0b013e3182194a25 PMC3197878

[17] Pollak CP, Stokes PE. Circadian rest-activity rhythms in demented and nondemented older community residents and their caregivers. J Am Geriatr Soc. 1997;45(4):446–52. 10.1111/j.1532-5415.1997.tb05169.x. 9100713 10.1111/j.1532-5415.1997.tb05169.x

[18] Smagula SF, Krafty RT, Taylor BJ, Martire LM, Schulz R, Hall MH. Rest-activity rhythm and sleep characteristics associated with depression symptom severity in strained dementia caregivers. J Sleep Res. 2017;26(6):718–25. 10.1111/jsr.12549. 28488270 10.1111/jsr.12549 PMC5681446

[19] Tranah GJ, et al. Circadian activity rhythms and risk of incident dementia and MCI in older women. Ann Neurol. 2011;70(5):722–32. 10.1002/ana.22468. 22162057 10.1002/ana.22468 PMC3244839

[20] Hoyos CM, et al. Circadian rhythm and sleep alterations in older people with lifetime depression: a case-control study. BMC Psychiatry. 2020;20(1): 192. 10.1186/s12888-020-02606-z. 32349697 10.1186/s12888-020-02606-z PMC7191686

[21] Chellappa SL, Vujovic N, Williams JS, Scheer FAJL. Impact of circadian disruption on cardiovascular function and disease. Trends Endocrinol Metab TEM. 2019;30(10):767–79. 10.1016/j.tem.2019.07.008. 31427142 10.1016/j.tem.2019.07.008 PMC6779516

[22] Buysse DJ, Reynolds CF, Monk TH, Berman SR, Kupfer DJ. The Pittsburgh sleep quality index: a new instrument for psychiatric practice and research. Psychiatry Res. 1989;28(2):193–213. 10.1016/0165-1781(89)90047-4. 2748771 10.1016/0165-1781(89)90047-4

[23] Kreutz C, Müller J, Schmidt ME, Steindorf K. Comparison of subjectively and objectively assessed sleep problems in breast cancer patients starting neoadjuvant chemotherapy. Support Care Cancer. 2021;29(2):1015–23. 10.1007/s00520-020-05580-0. 32556623 10.1007/s00520-020-05580-0 PMC7767899

[24] Lee H-A, et al. Comparison of wearable activity tracker with actigraphy for sleep evaluation and circadian rest-activity rhythm measurement in healthy young adults. Psychiatry Investig. 2017;14(2):179. 10.4306/pi.2017.14.2.179. 28326116 10.4306/pi.2017.14.2.179 PMC5355016

[25] Czeisler CA, et al. Stability, precision, and near-24-hour period of the human circadian pacemaker. Science. 1999;284(5423):2177–81. 10.1126/science.284.5423.2177. 10381883 10.1126/science.284.5423.2177

[26] Klerman EB, Gershengorn HB, Duffy JF, Kronauer RE. Comparisons of the variability of three markers of the human circadian pacemaker. J Biol Rhythms. 2002;17(2):181–93. 10.1177/074873002129002474. 12002165 10.1177/074873002129002474

[27] Rösler L, van der Lande G, Leerssen J, Cox R, Ramautar JR, van Someren EJW. Actigraphy in studies on insomnia: worth the effort? J Sleep Res. 2023;32(1): e13750. 10.1111/jsr.13750. 36217775 10.1111/jsr.13750 PMC10078209

[28] Jeon SY, et al. Circadian rest-activity rhythm and longitudinal brain changes underlying late‐life cognitive decline. Psychiatry Clin Neurosci. 2023;77(4):205–12. 10.1111/pcn.13521. 36527292 10.1111/pcn.13521 PMC10360409

[29] De Zambotti M, Goldstone A, Claudatos S, Colrain IM, Baker FC. A validation study of fitbit charge 2^™^ compared with polysomnography in adults. Chronobiol Int. Apr.2018;35(4):465–76. 10.1080/07420528.2017.1413578. 29235907 10.1080/07420528.2017.1413578

[30] Haghayegh S, Khoshnevis S, Smolensky MH, Diller KR, Castriotta RJ. Accuracy of wristband Fitbit models in assessing sleep: systematic review and meta-analysis. J Med Internet Res. 2019;21(11): e16273. 10.2196/16273. 31778122 10.2196/16273 PMC6908975

[31] Benedetti D, et al. Heart rate detection by Fitbit ChargeHR ^TM^: A validation study versus portable polysomnography. J Sleep Res. 2021;30(6):e13346. 10.1111/jsr.13346. 33837981 10.1111/jsr.13346 PMC9286609

[32] Bowman C, et al. A method for characterizing daily physiology from widely used wearables. Cell Rep Methods. 2021;1(4):100058. 10.1016/j.crmeth.2021.100058. 34568865 10.1016/j.crmeth.2021.100058 PMC8462795

[33] Wu F, Langer P, Shim J, Fleisch E, Barata F. Comparative efficacy of commercial wearables for circadian rhythm home monitoring from activity, heart rate, and core body temperature. IEEE J Biomed Health Inform. 2025;29(2):900–8. 10.1109/JBHI.2024.3471254. 39348247 10.1109/JBHI.2024.3471254

[34] Cho C-H, Lee T, Kim M-G, In HP, Kim L, Lee H-J. Mood prediction of patients with mood disorders by machine learning using passive digital phenotypes based on the circadian rhythm: prospective observational cohort study. J Med Internet Res. 2019;21(4): e11029. 10.2196/11029. 30994461 10.2196/11029 PMC6492069

[35] Jeong S, et al. Circadian rhythm of heart rate assessed by wearable devices tends to correlate with the circadian rhythm of salivary cortisol concentration in healthy young adults. Chronobiol Med. 2020;2(3):109–14. 10.33069/cim.2020.0022.

[36] Peng H-L, Lorenz RA, Chang Y-P. Factors associated with sleep in family caregivers of individuals with dementia. Perspect Psychiatr Care. 2019;55(1):95–102. 10.1111/ppc.12307. 29971795 10.1111/ppc.12307

[37] Albert MS, et al. The diagnosis of mild cognitive impairment due to Alzheimer’s disease: recommendations from the National Institute on Aging-Alzheimer’s Association workgroups on diagnostic guidelines for Alzheimer’s disease. Alzheimers Dement J Alzheimers Assoc. 2011;7(3):270–9. 10.1016/j.jalz.2011.03.008. 10.1016/j.jalz.2011.03.008 PMC3312027 21514249

[38] Lee DY, et al. A normative study of the mini-mental state examination in the Korean elderly. J Korean Neuropsychiatr Assoc. 2016;41(3):508–25.

[39] Sohn SI, Kim DH, Lee MY, Cho YW. The reliability and validity of the Korean version of the Pittsburgh sleep quality index. Sleep Breath Schlaf Atm. 2012;16(3):803–12. 10.1007/s11325-011-0579-9. 10.1007/s11325-011-0579-9 21901299

[40] Lee HS, Kim DK, Kim JH, Koh HJ, Koo HM, Kwon EJ. Measurement of stress in the caregivers of dementia patients: reliability and validity of the revised-memory and behavior problem checklist and the burden interview. Korean J Clin Psychol. 2004;23(4):1029–50.

[41] Zarit SH, Reever KE, Bach-Peterson J. Relatives of the impaired elderly: correlates of feelings of burden. Gerontologist. 1980;20(6):649–55. 10.1093/geront/20.6.649. 7203086 10.1093/geront/20.6.649

[42] Bae JN, Cho MJ. Development of the Korean version of the Geriatric Depression Scale and its short form among elderly psychiatric patients. J Psychosom Res. 2004;57(3):297–305. 10.1016/j.jpsychores.2004.01.004. 15507257 10.1016/j.jpsychores.2004.01.004

[43] Chun MY. Validity and reliability of korean version of international physical activity questionnaire short form in the elderly. Korean J Fam Med. 2012;33(3):144–51. 10.4082/kjfm.2012.33.3.144. 22787536 10.4082/kjfm.2012.33.3.144 PMC3391639

[44] Ainsworth BE, et al. Compendium of physical activities: an update of activity codes and MET intensities. Med Sci Sports Exerc. 2000;32(9):S498-504. 10.1097/00005768-200009001-00009. 10993420 10.1097/00005768-200009001-00009

[45] Hébert R, Bravo G, Préville M. Reliability, validity and reference values of the Zarit Burden Interview for assessing informal caregivers of community-dwelling older persons with dementia. Can J Aging Rev Can Vieil. 2000;19(4):494–507. 10.1017/S0714980800012484.

[46] Cornelissen G. Cosinor-based rhythmometry. Theor Biol Med Model. 2014;11(1): 16. 10.1186/1742-4682-11-16. 24725531 10.1186/1742-4682-11-16 PMC3991883

[47] McCurry SM, Logsdon RG, Teri L, Vitiello MV. Sleep disturbances in caregivers of persons with dementia: contributing factors and treatment implications. Sleep Med Rev. 2007;11(2):143–53. 10.1016/j.smrv.2006.09.002. 17287134 10.1016/j.smrv.2006.09.002 PMC1861844

[48] Gallagher VT, Reilly SE, Rossetti MA, Mattos M, Manning C. Factors associated with reduced sleep among spouses and caregivers of older adults with varying levels of cognitive decline. Psychogeriatrics. 2024;24(2):223–32. 10.1111/psyg.13064. 38098187 10.1111/psyg.13064 PMC11578025

[49] Gallagher V, et al. Insomnia symptoms among caregivers of persons with cognitive decline in an outpatient memory clinic. Sleep Med. 2024. 10.1016/j.sleep.2024.09.009. 39270597 10.1016/j.sleep.2024.09.009 PMC11663100

[50] Chang KJ, et al. Perceived sleep quality is associated with depression in a Korean elderly population. Arch Gerontol Geriatr. 2014;59(2):468–73. 10.1016/j.archger.2014.04.007. 24852666 10.1016/j.archger.2014.04.007

[51] Burkart S, Beets MW, Armstrong B, Hunt ET, Dugger R, von Klinggraeff L, et al. Comparison of multichannel and single-channel wrist-based devices with polysomnography to measure sleep in children and adolescents. J Clin Sleep Med. 2021;17(4):645–52. 33174529 10.5664/jcsm.8980 PMC8020711

[52] Fitbit Development. Heart Rate Time Series. Available: https://dev.fitbit.com/build/reference/web-api/heartrate-timeseries/. Accessed 16 May 2025.

