# Supplementary material for: Relationship between caregiving burden and alterations in circadian rhythms among spousal caregivers of individuals with cognitive impairment
Source: BMC Geriatr. 2025 Aug 23;25:652. doi: 10.1186/s12877-025-06316-7 (PMC12374363; doi:10.1186/s12877-025-06316-7)
Supplement: Supplementary file 1 — Additional file 1. Regression of the caregiving burden versus PSQI subdomain of SCGs (n=54). This table shows the results of a regression analysis between caregiving burden (measured by the Zarit Burden Interview) and various subdomains of sleep quality (assessed by the Pittsburgh Sleep Quality Index) in spousal caregivers. Additional file 2. Characteristics of Fitbit-derived sleep and circadian rhythm heart rate parameters by care recipient cognitive status (dementia vs. non-dementia). This table shows the results of comparative analysis of sleep and circadian rhythm parameters categorized by care recipients’ cognitive status (dementia vs. non-dementia caregivers) within the Fitbit group (n=54). [file 12877_2025_6316_MOESM1_ESM.docx]

Additional file 1. Regression of the caregiving burden versus PSQI subdomain of SCGs (n=54)

|  | **ZBI of SCGs** | | | |
| --- | --- | --- | --- | --- |
| *PSQI score* | **β** | **SE** | **t** | **p** |
| Total score | 0.089 | 0.026 | 0.596 | 0.554 |
| Sleep quality | 0.160 | 0.004 | 1.075 | 0.288 |
| Sleep latency | 0.049 | 0.005 | 0.319 | 0.751 |
| Sleep duration | 0.097 | 0.008 | 0.665 | 0.510 |
| Sleep efficiency | -0.052 | 0.009 | -0.353 | 0.726 |
| Sleep disturbance | 0.040 | 0.004 | 0.268 | 0.790 |
| Sleep medicine | -0.003 | 0.009 | -0.017 | 0.987 |
| Daytime function disturbance | 0.196 | 0.005 | 1.307 | 0.197 |

Notes: Covariates of the linear regression model include age and sex of SCGs and the cognitive level of care-recipients.

Abbreviations: SCG, spousal caregiver; ZBI, Zarit Burden Interview; PSQI, Pittsburgh Sleep Quality Index.
